# Patterns of availability and accuracy of risk factor data for cardiovascular diseases among people initiated on antiretroviral therapy at selected health facilities in Khomas region, Namibia: a retrospective, cross-sectional, quantitative study

**DOI:** 10.11604/pamj.2024.47.33.25340

**Published:** 2024-01-26

**Authors:** Roswitha Mahalie, Penehafo Angula, Kabwebwe Honoré Mitonga, Olanrewaju Oladimeji

**Affiliations:** 1 Faculty of Health and Applied Sciences, Namibia University of Science and Technology, Windhoek, Namibia,; 2 School of Public Health, Oshakati Campus, Faculty of Health Sciences, University of Namibia, Oshakati, Namibia,; 3 Department of Epidemiology and Biostatistics, School of Public Health, Sefako Makgatho Health Sciences University, Pretoria, South Africa

**Keywords:** Cardiovascular diseases, Khomas, HIV, PLHIV

## Abstract

**Introduction:**

quality data is a prerequisite for timely decision-making and measuring health outcomes in public health settings. Comorbidities such as cardiovascular diseases (CVDs) among people living with HIV (PLHIV), require a robust system that ensures credible data at all data-producing levels. The study at determining the level of availability and completeness of CVDs risk factors data of PLHIV.

**Methods:**

a quantitative study was conducted to extract CVDs risk factors data retrospectively from 529 patient care booklets (PCBs) between 2004 and 2017. The analysis was done with the Statistical Package for Social Sciences (SPSS) version 25. Pearson Chi-Square was used to test for associations. The level of significance was at p ≤ 0.05.

**Results:**

the study revealed that 72.8% of patients are at risk of CVDs due to incomplete demographics (73.72%) and other systemic data (41.18%). A significant association was found (Pearson Chi-Square test 19.907; p-value of 0.001) between average visits per year, accurate data recording, and active status of the patient. Lost to follow-up (15%) and true retention (27.2%) was significantly associated with the last Antiretroviral Therapy (ART) status of a patient (Pearson Chi-Square test 87.754; p-value of 0.001).

**Conclusion:**

the study that despite concerted efforts to improve data quality, the availability and completeness of data remain unsatisfactory. Lack of harmonised data screening and analysis efforts for CVDs risk factors is found to be a significant risk factor in ensuring integrated routine measuring of CVDs health outcomes for PLHIV.

## Introduction

Recent statistics show that non-communicable diseases (NCDs), in particular cardiovascular diseases, which are mainly driven by poverty, globalization of marketing and trade of health-harming products, rapid urbanization, and population growth are escalating disproportionately among low-income and lower-middle-income countries [1-3]. These risks have been rated as a growing public health concern in Africa, which has resulted in the multi-morbidity of risk of noncommunicable forms of CVDs, such as increased rates of hypertension, smoking, obesity, and initiation of antiretroviral therapy. The public health concern is especially critical in sub-Saharan Africa where an estimated 54% of people living with HIV (PLHIV) are currently on antiretroviral treatment (ART), of which 45% are virally suppressed [4]. To ensure evidence for program intervention, measuring achievements, sharing experiences, and making future projections, it is anticipated that data, information, and knowledge derived from well-established monitoring systems are subjected to sound data management processes. Examples of such processes include addressing gaps experienced with structural and functional data management systems; data-driven performance reviews; enabling environment whereby patients take ownership of their own health outcomes; and management of patient-level of data within community safety nets [5-7].

The World Health Organisation (WHO) reported that detailed high-quality data relating to the Sustainable Development Goals (SDGs) and the post-2015 development agenda are not routinely collected [8], thus resulting in the deficiency of regular computation of national levels and trends, or disaggregation across key dimensions of inequality [9]. Similarly, a study conducted by Dentler *et al*. revealed that unstructured data format, incorrect data items, and incomplete view of patient histories, lack of relations between data items, lack of detail and lack of standardisation were among the most common problems which resulted in the unreliable computation of specific indicators. The study further aligned these data quality issues against three different quality dimensions such as availability in a structured format, completeness, and correctness [10].

In the study conducted by Patel *et al*. in 2018, it is revealed that few NCD-HIV integrated programs with screening and management approaches exist in resource-limited settings, although the efficacy of such integration is not well-established [11]. This same notion is further cemented by Magodoro *et al*. who recommended the integration of non-communicable disease care and active screening into routine HIV care [12]. In addition, Nduka *et al*. also agree that HIV-infected patients commencing antiretroviral treatment in these settings may require regular hypertension screening and other cardiovascular risk assessments [13].

The HIV/AIDS-related deaths followed by stroke are the top 10 leading causes of death in Namibia [14,15]. Nevertheless, combating the epidemiological transition in Namibia, high-quality data, and effective data quality assessment are required for accurately evaluating the impact of health interventions and measuring health outcomes [16]. A robust Health Information System (HIS) is needed to ensure credible data at all data-producing levels. These data elements ought to be captured, analysed, and reported in robust HIS, which is an essential backbone for monitoring, tracking, and solving important health issues [17]. Amidst the eutopia of adhering to all the criteria of data management at any health facility, data quality issues remain a constant challenge in Namibia [9].

Although data CVD risk factors are routinely collected at ART clinics, it is evident that such data is not accurately recorded at health facilities in the Khomas health district. Hence, this article aimed at determining the level of availability, and completeness of data related to CVDs and their associated risk factors by reviewing health facility-based data obtained from individual patient care booklets at selected health facilities in the Khomas region.

## Methods

**Study design:** this study adopted a retrospective, cross-sectional, quantitative study design to purposively select targeted primary healthcare facilities to determine the level of data availability and completeness in the Khomas region.

**Setting:** an audit trail on data availability and completeness was performed at selected health facilities such as Robert Mugabe, Otjomuise, Maxuilili, Donkerhoek, Groot Aub, Hakahana, and Wanaheda clinics situated in the Khomas health district in Central Namibia between March 2019 and April 2020.

**Participants:** data were randomly selected and extracted from the ePMS. Subsequently, a data audit trail was done by comparing data elements captured in the ePMS with those in PCBs.

**Variables:** completeness of data is crucial to determine exposure emanating from CVDs risk factors among PLHIV initiated on ART; hence missing data was identified as the predominant variable in this study to avoid misleading conclusions.

**Data sources/measurement:** a tailor-made quantitative data extraction checklist was used. Individual patient care booklets and ePMS in which key CVD-related data is recorded at every follow-up visit were randomly selected to conduct a data trail. Data was thereafter extracted from PCBs and ePMS. A completeness score that followed a similar approach and process as Xiao *et al*. [16], had been developed to determine percentages of recordkeeping and missing data at each of the selected health facilities.

**Bias:** there was no need to measure reliability and validity for secondary data. The extraction of data from both ePMS and PCBs was conducted by the same individual to minimise misclassification or information bias which is a common phenomenon in retrospective studies.

**Study size:** seven (7) out of the twelve (12) health facilities were selected to extract data from 529 PCBs.

**Inclusion and exclusion criteria:** several factors such as the availability of the electronic patient monitoring system (ePMS) and the availability of the custodians of data at peripheral levels influenced a site's likelihood of being selected for the data quality exercise. Clients enrolled in the program before January 2004 and after 2017 were excluded from the study.

**Quantitative variables:** critical data elements such as blood pressure, weight, height, smoking, and alcohol history are captured in PCBs once PLHIV comes for screening at health facilities. Furthermore, data availability and completeness for variables such as marital status; date of birth, age; start ART and start the year; the total average of follow-up days; and the overall average of visits per year were considered. Cumulative assessments of those clients who were transferred out, lost to follow-up, or who have died quarterly were also considered.

**Statistical methods:** the extracted data were entered into IBM (R) SPSS version 26 to perform statistical analyses. Descriptive statistics were used to categorise and describe continuous variables that were summarised using frequency distributions. Bivariate and multivariate analyses were performed by constructing two by two tables for each category. Pearson Chi-Square was used to test for associations. The level of significance was at p ≤ 0.05. Findings were presented as tables and bar charts. Socio-demographic characteristics were tabulated by age and sex.

**Ethical consideration:** the study was approved by the Research and Ethics Committee of the University of Namibia and the Ministry of Health and Social Services. The study conforms with the principles outlined in the Declaration of Helsinki. Furthermore, confidentiality as well as anonymity were maintained.

## Results

**Participants:** in total seven (7) out of the twelve (12) health facilities were selected to extract data from five-hundred and twenty-nine (529) PCBs.

**Descriptive data:** in Table 1 the date of birth (DOB) and sex distribution of studied subjects emanated from the 529 records extracted for PLHIV initiated on ART, indicates 34.40% males whilst 51.42% females with a median ±SD age of 38.10 years. From this data, 0.14% of records did not indicate the DOB of the studied subjects. The findings revealed that a total of 72.8% of patients are at risk due to inconsistent, incomplete, or missing demographic data (marital status, DOB, and age) and other systemic data (start ART year; total average of follow-up days; total average of visits per year). Results show that 97.1% of data elements extracted from the client records were missing or incomplete in urban areas at a rating score of 25% compared to 2.9% in the rural areas. In a nutshell, 96.4% of these records rated at 75% are missing or incomplete in urban areas as opposed to 3.6% in rural areas. The Pearson Chi-Square test (2.204, a p-value of 0.138; likelihood ratio test 2.205, a p-value of 0.155) shows no significant difference between missing data in rural or urban health facilities in terms of availability and completeness of the selected data elements in Table 2.

**Table 1:** date of birth and sex distribution of studied subjects

Classification by DOB	Male		Female		Total	
	No.	%	No.	%	No.	%
2010-2017	3	0.57	6	1.13	9	0.02
2000-2009	1	0.19	3	0.57	4	0.01
1990-1999	16	3.02	69	13.04	85	0.16
1980-1989	58	10.96	103	19.47	161	0.30
1970-1979	72	13.61	71	13.42	143	0.27
1960-1969	23	4.35	15	2.84	38	0.07
1950-1959	7	1.32	4	0.76	11	0.02
1940-1949	2	0.38	1	0.19	3	0.01
**Missing data (DOB)**					**75**	**0.14**
Total count	182	34.40	272	51.42	529	100.00

DOB: date of birth

**Table 2:** urban and rural distribution of cardiovascular diseases risk factors for people living with HIV initiated on antiretroviral therapy

Percentage missing * urban/rural crosstabulation	Rural	Urban	Total
25% within percentage missing	2.9%	97.1%	385
25% within urban/rural	57.9%	73.3%	100.0%
Count	11	374	100.0%
75% within percentage missing	5.6%	94.4%	144
75% within urban/rural	42.1%	26.7%	100.0%
Count	8	136	72.8%
Total percentage within percentage missing	3.6%	96.4%	529
Total percentage within urban/rural	100.0%	100.0%	100.0%
Total count	19	510	27.2%

Sig. 2-sided: Pearson Chi-Square 0.138; likelihood ratio 0.155

**Figure 1:**
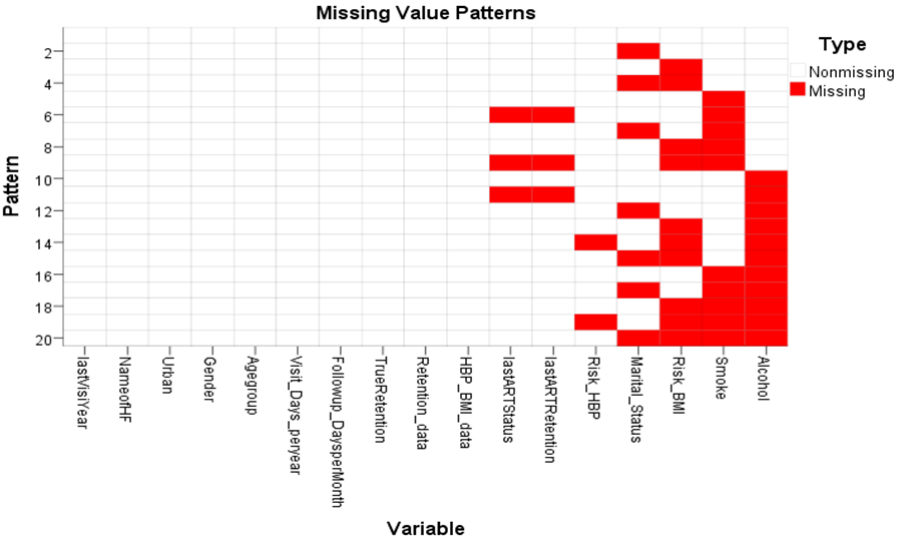
missing value patterns of cardiovascular diseases risk factor data among people living with HIV initiated on antiretroviral therapy

Outcome data/main results: the study revealed that not all parameters (blood pressure, pulse, respiration, weight, height, and respiration) of patients are captured or recorded in the PCB and subsequently in the ePMS. The study also revealed that 73.72% missing values in ePMS, while 41.18 % of the variables were incomplete. The missing value patterns were more inclined toward alcohol, smoking, and those at risk of increased body mass index (BMI) as presented in Figure 1. A lower concentration is observed in the last ART status and high blood pressure. Non-missing variable patterns are mostly observed in sex, age, and average follow-up days.

Furthermore, results showed that for patients who had an average of 1-3 visits per year, 38.00% were active yet had their records were poorly managed at the clinic as indicated in Table 3. A total of 45.50% of the patients with 6-7 visit days per year were also presented with poor records management. However, 62% of those with properly managed data had at least 1-3 days visit per year compared to 79.20% who visited the health facility at least 4-5 days per year. In addition, 54.50% of patients who visited the health facility at least 6-7 days per year had good-quality records. There was a significant association found between average visits per year, poor data recording, and active status of the patient (Pearson Chi-Square test at 19.907, a p-value of 0.001).

**Table 3:** average visits per year of people living with HIV initiated on antiretroviral therapy and quality of data recorded

Visit per year of PLHIV initiated on ART	Quality of data recorded on active status of PLHIV initiated on ART		
	Good recordkeeping	Poor recordkeeping	Total
**Day 1 to 3**			
Count	176	108	284
Percentage within visit per year	62.00%	38.00%	100.00%
**Day 4 to 5**			
Count	168	44	212
Percentage within visit per year	79.20%	20.80%	100.00%
**Day 6 to 7**			
Count	18	15	33
Percentage within visit per year	54.50%	45.50%	100.00%
Total Count	362	167	529
% within visit per year	68.40%	31.60%	100.00%

Sig. 2-sided: Pearson Chi-Square 0.001; likelihood ratio 0.001; PLHIV: persons living with HIV; ART: antiretroviral therapy

The findings of the study show that an alarming number of 53.80% of patients whose records were poorly recorded and managed whilst active on ePMS were lost to follow-up. It is further revealed that 15.00% of those who were still actively on treatment had poorly managed records. However, 85.00% of those who were still on treatment and 46.20% of those who were lost to follow-up had properly managed records. The Pearson Chi-Square test (105.885) and the Likelihood ratio (106.165) both show a p-value of 0.001, which implies a significant association between data recording and with the last ART status risk of the patient.

**Other analyses:** the association between retention of patients and average follow-up days risk per year, last ART status, and data recording risk and among PLHIV in the Khomas health district is presented in Table 4. The findings of the study show that a total of 52.90% of patients whose records were poorly recorded are indicated as lost to follow-up compared to 22.70% who are still active in the system although their records were poorly managed. Similar to the previous finding, data recording is significantly associated with the retention of patients together with poor records management (Pearson Chi-Square test 46.191; p-value of 0.001).

**Table 4:** analysis of true retention and average follow-up days of people living with HIV initiated on antiretroviral therapy

Follow-up days of PLHIV initiated on ART	True Retention of PLHIV initiated on ART		
	Active	Lost	Total
**Days 30 and below**			
Count	28	24	52
Percentage within follow-up days	53.80%	46.20%	100.00%
**Days 31 to 60**			
Count	117	67	184
Percentage within follow-up days	63.60%	36.40%	100.00%
**Days 61 and above**			
Count	229	64	293
Percentage within follow-up days	78.20%	21.80%	100.00%
Total count	374	155	529
Percentage within follow-up days	70.70%	29.30%	100.00%

Sig. 2-sided: Pearson Chi-Square 0.001; likelihood ratio 0.001; PLHIV: persons living with HIV; ART: antiretroviral therapy

The results obtained in the current study revealed that 46.20% of the patients who were lost for 30 days and below were not adequately retained in ePMS and therefore potentially at risk for CVDs. Furthermore, 78.20% of the patients who had an average of days and above are still active on the system. The Pearson Chi-Square test (19.489) and the Likelihood ratio (19.203) both showed a p-value of 0.001, which implied that the researcher rejected the null hypothesis. Therefore, the researcher concluded with 99.99% confidence that there was a significant association between a PLHIV initiated on ART who are retained in ePMS and average follow-up days. The findings revealed that 85% of the patients who are on treatment are still active as per their last ART status while 15% are lost and likely at risk. True retention was significantly associated with the last ART status of a patient (Pearson Chi-Square test (87.754) and the Likelihood ratio (86.320) both showed a p-value of 0.001).

Finally, the study findings revealed that 82.10% of active clients have their records poorly managed of which 17.90% are indicated as lost. Another 66.10% are presented with an active status and have their records well managed. However, 33.90% of patients' good records are reportedly lost from ePMS. The Pearson Chi-Square test (13.303 with a p-value of 0.001) showed implied a significant association between true retention and the quality of data recording practices.

## Discussion

**Key findings:** the study was conducted to determine the level of availability and completeness of CVDs risk factors data of PLHIV at selected health facilities in Khomas region, Namibia. The study found that missing (73.72%) and incomplete (41.18%) data of priority variables posit to be major contributing factors to the current challenges of getting accurate information on CVDs among PLHIV initiated on ART. Furthermore, of patients who had an average of 1-3 visits per year, 38.00% were active yet had their records are poorly managed at the clinic Over fifty percent (52.90%) of patients whose records were poorly recorded are indicated as lost to follow-up compared to 22.70% who are still active in the system although their records were poorly managed (Pearson Chi-Square test 46.191; p-value of 0.001).

**Interpretation:** the study found that the overall rate of missing (73.72%) and incomplete (41.18 %) data are major contributing factors to inaccurate health information. These challenges ought to be addressed systematically. The findings of this study are consistent with studies that postulated that completeness, consistency, and accuracy of data of each patient is a critical criterion for examining data quality issues [18-21]. There is a need to improve the completeness of death registration and the quality of the cause of death information as emphasised in a study conducted by Bradshaw *et al*. [22]. A similar observation was made by Opare J *et al*. who revealed that data completeness, provision of feedback, and advocacy for public health intervention need strengthening for non-communicable diseases surveillance. This same study of Opare *et al*. found that at least 60% of data for NCDs were complete in the eastern region of Ghana [23]. Furthermore, Sanuade *et al*. also suggested that the documentation of patient characteristics such as socioeconomic and demographic characteristics may serve as useful tools that will intensify epidemiologic surveillance of diseases such as CVDs [24].

The authors concur that complete data and accurate record management are essential in ensuring that targeted health services are provided to PLHIV initiated on ART. Average visits per year, and poor data recording were found to be significantly associated to the active status of the patient (Pearson Chi-Square test at 19.907, a p-value of 0.001). In this study, results derived from the 45.50% of the patients with 6-7 days visit per year exhibited patterns of poor records management compared to 54.50% of patients who had good quality records.

In this study, LTFU seems not to be a major challenge since 82.10% of active PLHIV have their records poorly managed of which only 17.90% are indicated as lost. On the contrary, a study done at Mulanje Mission Hospital, Malawi revealed that 31.61% of patients had incomplete or missing ART cards, resulting in 68.38% lost to follow-up (LTFU) patient´s data that could be analysed [25]. Similarly, a retrospective study in Guinea-Bissau found an overall incidence rate of 51.1 per 100 person-years who had been lost to follow-up in the ART programme [26].

Institutionalised reporting requirements would likely reduce the incidence of incompleteness, inconsistency, and inaccuracy of data at the health facility level. Adopting a strong feedback mechanism such as a quarterly data review sessions coupled with an action-oriented approach whereby findings will be discussed, best practices are shared and challenges about data quality issues are discussed at the different levels of data consolidation, synthesis, and use is recommended by the authors.

General data management practices which were mostly inclined towards delivering health interventions for communicable diseases, hence robust systems capturing data on major non-communicable diseases among PLHIV were not readily available were considered as a potential limitation in this study. This led to an underestimate of the relationship between the level of availability and completeness of data of co-morbid PLHIV. The main strength of this study is strong paper-based systems that are still in place despite the fact that these systems ought to be aligned with interoperable electronic systems.

## Conclusion

The study concluded that routine data collection efforts to determine the prevalence of health risk factors for CVDs are routinely done for all ART clients at health facilities. However, missing (73.72%) and incomplete (41.18 %) data are major challenges that contribute to inaccurate information. Average visits per year, poor data recording, and active status of the patient were found to be significant risks to an integrated routine measuring of CVDs health outcomes for PLHIV.

### What is known about this topic

- *Data quality assessments are regularly advocated for;*
- *Data management practices are mostly inclined towards delivering health interventions for communicable diseases;*
- *Comorbidity of NCDs among PLHIV*.

### What this study adds

- *The study unearthed that missing and incomplete data are contributing challenges to a lack of integrated routine measuring of CVDs health outcomes for PLHIV;*
- *The study findings justify the development of a feedback mechanism that includes quarterly action-oriented data review sessions, interoperability of information from the lowest level of service delivery;*
- *Institutionalisation of reporting requirements aimed at reducing the occurrence of incompleteness, inconsistency, and inaccuracy of data at the health facility level*.
